# Normative data of contact heat evoked potentials from the lower extremities

**DOI:** 10.1038/s41598-018-29145-8

**Published:** 2018-07-20

**Authors:** J. Rosner, P. Hostettler, P. S. Scheuren, L. Sirucek, J. Rinert, A. Curt, J. L. K. Kramer, C. R. Jutzeler, M. Hubli

**Affiliations:** 1 Spinal Cord Injury Center, Balgrist University Hospital, University of Zurich, Zurich, Switzerland; 2 International Collaboration on Repair Discoveries (ICORD), University of British Columbia, Vancouver, British Columbia Canada; 3 School of Kinesiology, University of British Columbia, Vancouver, British Columbia Canada

## Abstract

Contact heat evoked potentials (CHEPs) have become an acknowledged research tool in the assessment of the integrity of the nociceptive system and gained importance in the diagnostic work-up of patients with suspected small fiber neuropathy. For the latter, normative values for CHEP amplitude and latency are indispensable for a clinically meaningful interpretation of the results gathered in patients. To this end, CHEPs were recorded in 100 healthy subjects over a wide age range (20–80 years) and from three different dermatomes of the lower extremities (L2, L5, and S2). A normal baseline (35–52 °C) and increased baseline stimulation (42–52 °C) were applied. Statistical analysis revealed significant effects of stimulation site, stimulation intensity, and sex on CHEP parameters (N2 latency, N2P2 amplitude, and NRS). Significant positive correlations of body height with N2 latency, and pain ratings with N2P2 amplitudes were observed. This is the first time that normative values have been obtained from multiple dermatomes of the lower extremities. The present dataset will facilitate the clinical application of CHEPs in the neurophysiological diagnosis of small fiber neuropathy and by discerning pathological findings help establish a proximal-distal gradient of nerve degeneration in polyneuropathies.

## Introduction

Contact heat stimulation activates small diameter A-delta and C fiber nociceptors within the epidermis^1,2^. The recorded cortical potential is related to conduction within peripheral A-delta fibers, relayed to central spinothalamic projections, thalamus and cortex^3–5^. Contact heat evoked potentials (CHEPs) have been employed to document damage along the entire nociceptive neuraxis in a wide range of neurological diseases^6–10^.

The use of CHEPs in the neurophysiological assessment of disorders affecting the lower extremities has been challenged by a poor signal-to-noise ratio and technical drawbacks^2,11,12^. While several studies have reported normative values of CHEPs from the upper extremities^11,13–15^, few have addressed the lower extremity^12^.

The availability of such normative data may help close an important diagnostic gap in increasingly prevalent conditions, such as small fiber neuropathies^16^. Small fiber pathologies often pose a challenge as conventional neurophysiology does not yield conclusive results^17,18^.

The effect of stimulus intensity on the acquisition of CHEPs from the lower extremities has only recently been assessed systematically^12^. In that study, we demonstrated the superiority of the increased baseline (IB) protocol (42–52 °C) for the acquisition of CHEPs from lower extremities with higher signal persistence^12^. However, normative values for the lower extremities only exist for the normal baseline (NB) protocol (35–51 °C)^11,13^.

A recent multicenter study provides a large data set for commonly used stimulation sites^11^. However, the study protocol only included one site from the lower extremities, thus precluding its use for the assessment of length-dependency in polyneuropathies. As small fiber neuropathies often present in a distal-symmetrical fashion owing to a length-dependency of fiber degeneration^19^, normative values from proximal and distal sites are needed in order to establish a neurophysiological gradient.

With the IB protocol being the preferable stimulation paradigm for CHEP acquisition from the lower extremities^12^, the need for a comprehensive set of normative values for both stimulation protocols is addressed here. Beyond the proof of feasibility, the present study provides normative values across a wide range of age groups. In particular, the inclusion of an older population is of high clinical relevance, as this cohort is epidemiologically most often affected by polyneuropathies. As age already has a physiological impact on CHEP parameters, a robust and reliable stimulation paradigm (i.e., IB stimulation) is a requisite for a diagnostically meaningful approach.

## Material and Methods

### Subjects

 Hundred healthy subjects (47 men and 53 women) from three predefined age groups (20–40, 41–60, and 61–80 years) were included. Inclusion criteria were native language either English or German. Exclusion criteria included pregnancy, intake of psychoactive medication, and any neurological condition.

All participants provided written informed consent prior to the assessments and all procedures described below were in accordance with the Declaration of Helsinki. The study has been approved by the local ethics board ‘Kantonale Ethikkommission Zürich, KEK’ (EK-04/2006, PB_2016-02051, clinicaltrial.gov number: NCT02138344).

### Study design

**Figure 1:**
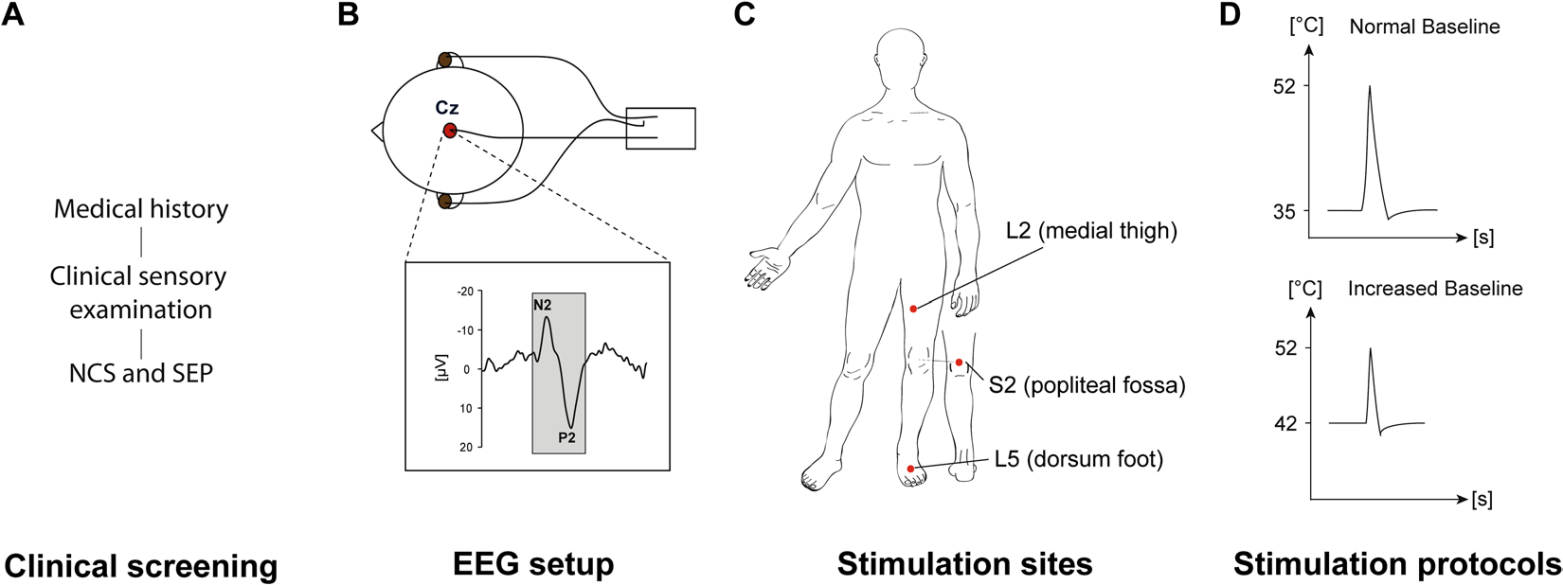
Summary of the study design: (**A**) clinical screening block including medical history, clinical sensory examination, somatosensory evoked potential (SEP), and nerve conduction study (NCS). (**B**) EEG setup for the CHEPs recording. (**C**) Stimulation sites of the CHEPs thermode. (**D**) Illustration of both stimulation protocols.

Subjects medical history was assessed and subsequently nerve conduction as well as somatosensory evoked potentials were recorded in order to exclude neuropathy. All subjects underwent a clinical sensory examination of mechanoreception and nociception, both of which were semi-quantitatively assessed according to the grading system of the International Standards for Neurological Classification of Spinal Cord Injury^20^. Afterwards subjects lay down in a supine position and three stimulation sites from the lower extremity were examined: the L2 dermatome at the inner side of the thigh, the L5 dermatome at the dorsum of the foot, and the S2 dermatome 5 cm above the popliteal fossa (Fig. 1C). The order of the tested dermatome and body side was randomized for each subject. CHEPs were recorded employing two different stimulation protocols: (1) the conventional *normal baseline protocol (NB)* followed by (2) the *increased baseline protocol (IB)*. The two protocols differ by their applied baseline temperature, i.e., 35 °C for the normal and 42 °C for the IB protocol, while the peak temperature of 52 °C was the same for both protocols (Fig. 1D)^21,22^. A summary of the study protocol is illustrated in Fig. 1.

### Acquisition of CHEPs

The CHEPs measurement set-up has been published elsewhere^4,5,15,21,23^. Briefly, the acquisition of CHEPs was performed using a contact heat stimulator with the established PATHWAY Pain & Sensory Evaluation System (Medoc Ltd., Ramat Yishai, Israel). The thermode surface of 27 mm consists of a heating thermo-foil covered with a layer of thermo-conductive plastic. The nominal heating rate of the thermode is 70 °C/s (thermo-foil), with a cooling rate of 40 °C/s (peltier element).

Cortical potentials to the noxious heat stimuli were recorded with 9 mm Ag/AgCl cup electrodes filled with conductive adhesive gel. The recording sites on the scalp were prepared with Nuprep (D.O. Weaver & Co. Aurora, CO) and alcohol. Cup electrodes were positioned on the vertex (Cz) referenced to the earlobes (A1-A2) according to the 10–20 system (Fig. 1B). The vertex position is considered as the most reliable position to record N2 and P2 potentials^24^. All signals were sampled at 2000 Hz using a preamplifier (20000x, bandpass filter 1–300 Hz, ALEA Solutions, Zurich, Switzerland). Data were recorded with 100 ms pre-trigger and a one second post-trigger in a customized program based on LabView (V2.04 CHEP, ALEA Solutions, Zurich, Switzerland).

Prior to the CHEP recordings, a familiarization procedure comprising a heat stimulus at the contralateral leg was applied. Contact heat stimuli were applied with an inter-stimulus interval of 8–12 sec. After each stimulus the thermode was marginally repositioned within the tested area to avoid peripheral receptor fatigue and habituation^1^. In addition, cued by an auditory signal provided four seconds after heat stimulus, subjects were asked to rate the perceived intensity of each stimulus using a numeric rating scale (NRS) ranging from 0 (no pain) to 10 (worst pain imaginable). The verbal instructions for the subjects comprised the following points: keep eyes open and fix a point on the ceiling, remain relaxed and quiet during the assessment, rate the perceived heat stimulus after the auditory signal on a scale ranging from 0 (no pain) to 10 (worst pain imaginable).

### Data analysis and statistics

In both stimulation protocols, stimuli were applied with the goal of 15 artifact-free signals without exceeding the total number of 20 trials. Signals were visually analyzed and trials with obvious muscle or ocular artifacts were discarded. The remaining signals were averaged and the N2P2 amplitude was visually inspected by two independent examiners. The whole EEG analysis was performed using a customized program based on LabView (V2.04 CHEP, ALEA Solutions, Zurich, Switzerland).

R software (version 3.3.1) and SPSS software (version 16) for Windows was used to conduct all statistical analyses and generate the graphs. The data were tested for normal distribution using the Shapiro-Wilk test and by visually inspecting histograms and Q-Q plots. While N2 latencies and NRS were normally distributed, N2P2 amplitudes were not. Statistical significance was set at α < 0.05 and was adjusted for multiple comparisons using Tukey contrasts.

To establish normative values, descriptive statistics (i.e., mean and 95% CI) were calculated. Sex difference in body height was tested using an independent t-test.

The main effects of stimulation protocol (i.e., NB, IB), stimulation sites (i.e., L2, L5, & S2), and age group were investigated by building a linear mixed model with protocol site and age group as fixed factors and random subject effects. Post-hoc tests were used to examine differences in CHEP parameters between stimulation sites under both stimulation paradigms.

Exploration of the effects of subject demographics such as age and body height as well as perceived pain during testing on CHEP parameters was performed using pairwise Spearman correlations. An additional general linear mixed model was set up to test the effect of sex and height on CHEP parameters. N2 latency, N2P2 amplitude, and NRS were set as dependent variables, while sex was included as a fixed factor and height as covariate. Examination of model diagnostics, in particular residuals of dependent variables, indicated that a logarithmic (log) transformation for amplitude data was necessary to meet model requirements for both general linear mixed models used.

## Results

### Subjects

A total of 100 healthy subjects participated in the study. Three had to be excluded due to the results of the clinical screenings, i.e., suspected neurological condition. The remaining subjects included 45 men and 52 women with a mean age of 47.6 ± 17.2 years. The subjects had a mean height of 171.6 ± 8.7 cm and men were significantly taller than women (p < 0.001).

### Main effects of stimulation protocol, stimulation site and age on CHEP parameters

**Figure 2:**
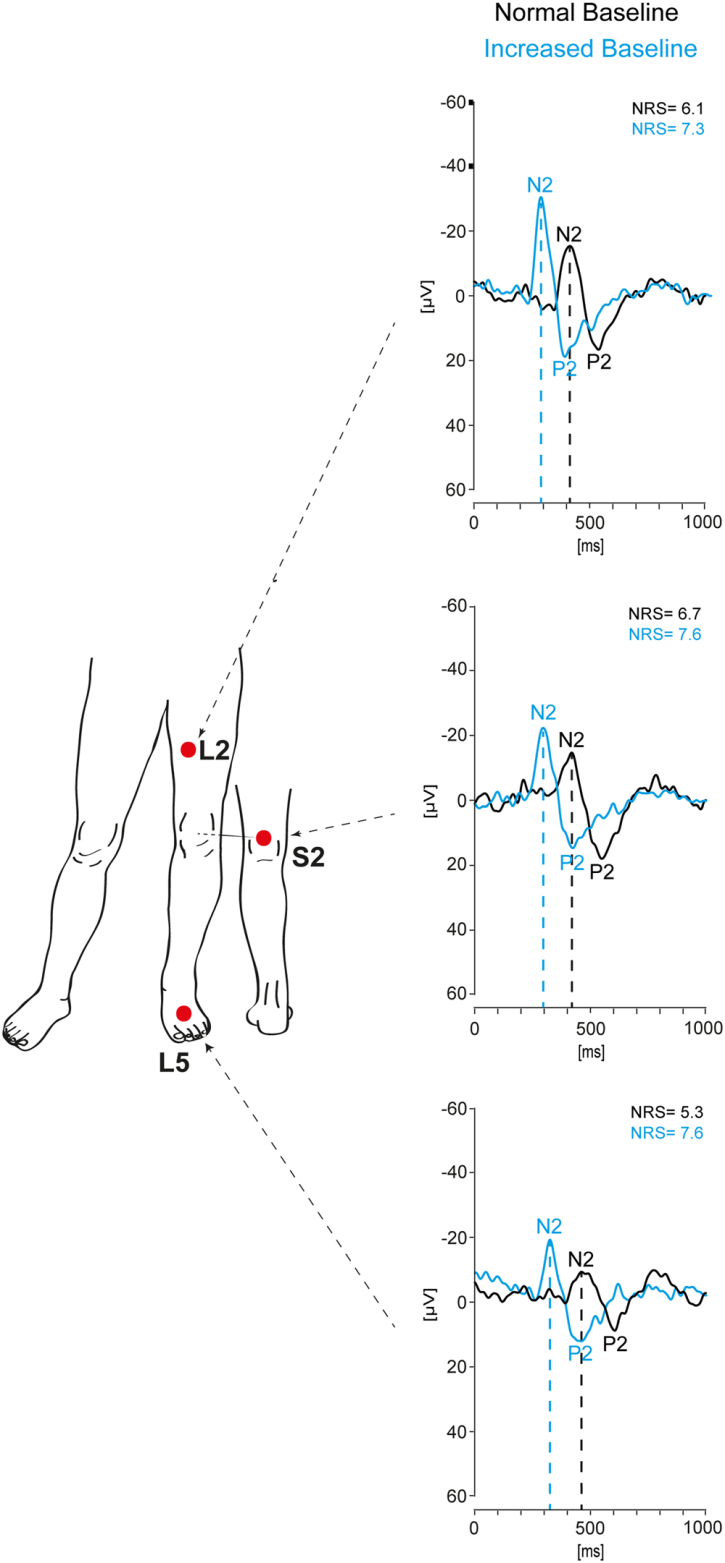
Representative example of CHEPs recordings from the lower extremities (female, 51 years) using the normal and increased baseline protocol for each tested site (L2, S2 & L5). Averaged signals of the normal baseline protocol are displayed in black, while averaged signals of the increased baseline protocol are shown in blue.

The dataset of 97 included subjects was used to establish normative values for CHEPs for lower extremities. Figure 2 illustrates a representative example of averaged CHEP signals for all three tested sites and both stimulation protocols.

The normative values (mean ± 95% CI) of the investigated parameters (N2 latencies, N2P2 amplitudes, and pain ratings (NRS)) for each stimulation site, stimulation protocol and age group are summarized in Table 1. The middle-aged and the elderly subject group showed significantly longer latencies and smaller amplitudes compared to the young group (see Table 2).

**Table 1:** CHEPs parameters (N2 latency, N2P2 amplitude & pain rating (NRS)) for each stimulation site (L2, L5 & S2) and for both protocols (normal baseline & increased baseline) displayed as mean ± 95% CI (only +95% CI for N2 latency) and the number of analyzed subjects (N) in each subgroup.

		young (20–40 yrs)				middle (41–60 yrs)				elderly (61–80 yrs)			
		N	*Normal Baseline*		*Increased Baseline*	N	*Normal Baseline*	N	*Increased Baseline*	N	*Normal Baseline*	N	*Increased Baseline*
L2	N2 lat [ms]	33	400 [412]	33	296 [306]	31	415 [430]	31	306 [320]	24	406 [422]	30	304 [320]
	N2P2 amp [µV]	33	33.7 [27.6, 39.8]	33	43.4 [36.7, 50.2]	31	19.8 [16.8, 22.8]	31	28.7 [24.5, 32.8]	24	20.6 [16.2, 25.1]	30	23.2 [17.8, 28.6]
	NRS	34	5.2 [4.6, 5.8]	33	6.8 [6.2, 7.4]	33	5.3 [4.5, 6.0]	31	6.7 [5.9, 7.5]	30	5.8 [5.2, 6.4]	30	6.9 [6.3, 7.5]
L5	N2 lat [ms]	31	442 [456]	34	331 [345]	27	455 [475]	29	356 [373]	20	478 [500]	27	362 [381]
	N2P2 amp [µV]	31	27.3 [23.0, 31.7]	34	34.13 [28.6, 39.5]	27	18.7 [15.9, 21.5]	29	26.9 [18.3, 35.4]	20	14.8 [11.9, 17.8]	27	19.9 [17.5, 22.3]
	NRS	34	4.0 [3.4, 4.6]	34	5.8 [5.1, 6.5]	33	4.3 [3.5, 5.1]	32	5.7 [4.8, 6.6]	20	3.8 [3.1, 4.5]	30	5.8 [5.1, 6.5]
S2	N2 lat [ms]	33	408 [420]	33	291 [298]	31	426 [442]	31	324 [337]	23	428 [443]	22	315 [332]
	N2P2 amp [µV]	33	34.4 [28.3, 40.5]	33	46.0 [39.1, 53.0]	31	22.8 [19.0, 26.6]	31	30.1 [25.5, 34.6]	23	25.0 [16.5, 33.5]	22	29.1 [23.0, 35.2]
	NRS	33	5.4 [4.7, 6.1]	33	6.9 [6.3, 7.5]	32	6.0 [5.3, 6.8]	31	7.0 [6.3, 7.7]	29	6.4 [5.7, 7.1]	26	7.4 [6.7, 8.1]

**Table 2:** Main effects of stimulation site, protocol and age group on CHEP parameters and multiple comparisons of stimulation sites.

	Main Effect						Multiple Comparisons				
	Protocol Effect		Dermatome Effect		Age Group Effect		Comparison	Normal Baseline		Increased Baseline	
	F	p	F	p	F	p		Estimate	p	Estimate	p
N2 lat [ms]	1817.0	<0.001	141.3	<0.001	4.7	<0.05	L5 vs. L2	+51	<0.001	+47	<0.001
							S2 vs. L2	+13	<0.01	+7	>0.05
							S2 vs. L5	−38	<0.001	−40	<0.001
N2P2 amp [log(µV)]	121.1	<0.001	31.7	<0.001	19.0	<0.001	L5 vs. L2	−0.18	<0.01	− 0.17	<0.001
							S2 vs. L2	+0.08	>0.05	+0.10	<0.05
							S2 vs. L5	+0.26	<0.001	+0.27	<0.001
NRS	304.9	<0.001	138.3	<0.001	0.2	>0.05	L5 vs. L2	−1.4	<0.001	−1.1	<0.001
							S2 vs. L2	+0.5	<0.01	+0.3	>0.05
							S2 vs. L5	+1.8	<0.001	+1.4	<0.001

**Figure 3:**
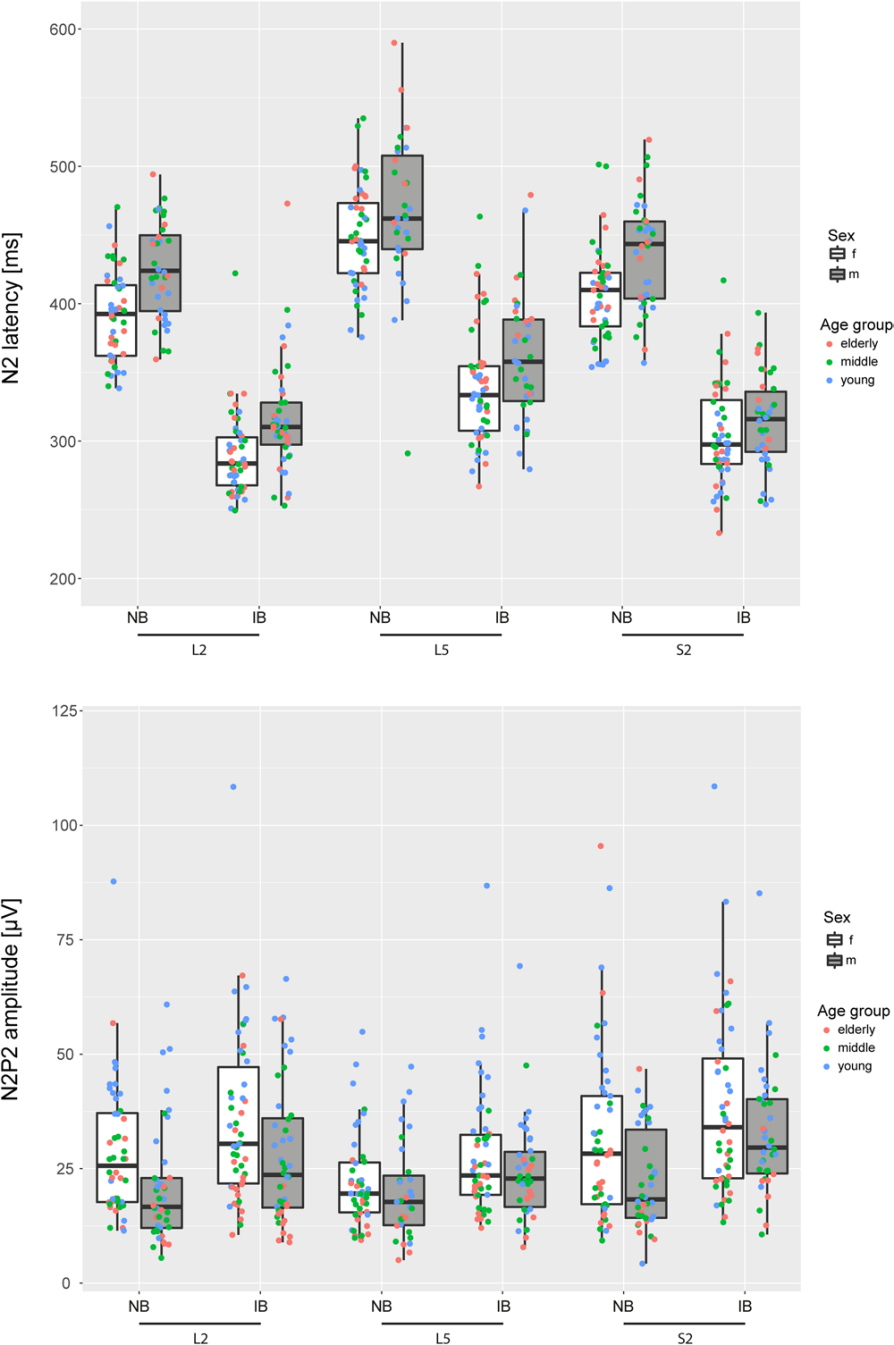
N2 latencies and N2P2 amplitudes for each stimulation site (L2, L5 & S2), both stimulation protocols (normal baseline (NB) & increased baseline (IB)), both sexes and three age groups. The subjects are color coded according to their age group.

Figure 3 shows N2 latencies and N2P2 amplitudes for each tested site, stimulation protocol, grouped by age and sex. The linear mixed model revealed significant main effects of stimulation protocol, site and age (groups) on all investigated CHEP parameters. Sex had no significant effect on N2 latencies (F = 1.0, p = 0.3), and NRS (F = 1.2, p = 0.3) when corrected for height. However, log(N2P2 amplitudes) were significantly higher in females compared to males (F = 4.9, p = 0.029). Further post-hoc tests primarily displayed significant differences between the L5 dermatome and the two further proximally located stimulation sites (L2 & S2), while the L2 and S2 dermatomes were comparable. Main effects and dermatome-wise comparisons are summarized in Table 2. In detail, the L5 dermatome featured longer N2 latencies and decreased N2P2 amplitudes.

### Correlations of age, body height, and perceived pain

**Figure 4:**
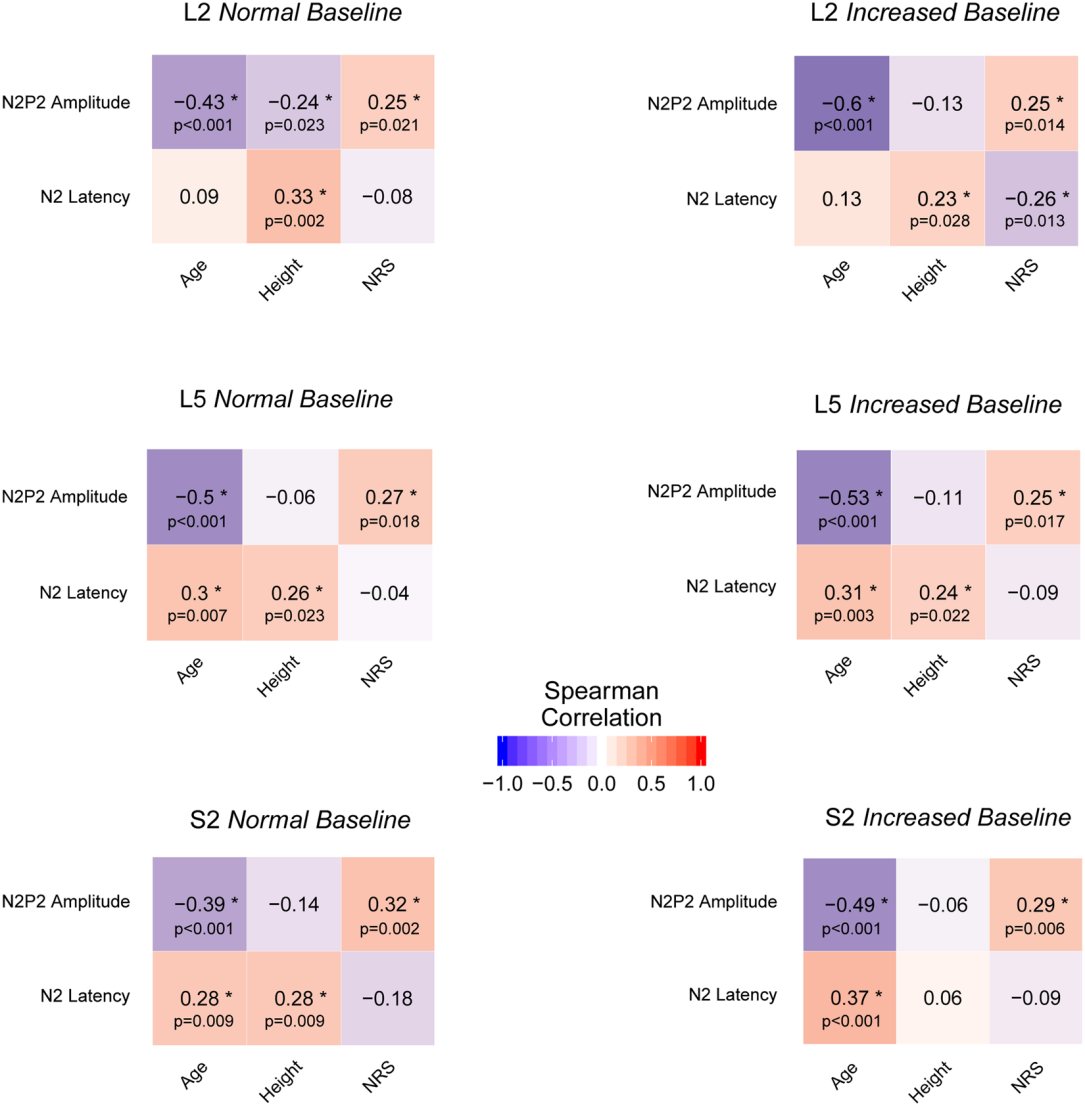
Spearman correlation matrices of CHEP parameters (N2P2 amplitudes & N2 latencies) and demographics for each stimulation site (L2, L5 & S2) and stimulation protocol (normal baseline & increased baseline). *Significant correlations.

The Spearman correlation analysis of CHEP latencies and amplitudes with age, body height, and pain ratings consistently disclosed significant negative correlations between age and N2P2 amplitudes (all p-values < 0.001). Significant positive correlations of age and N2 latencies emerged in the L5 and the S2 dermatome in both protocols (p < 0.01 in L5 NB, L5 IB & S2 NB; p < 0.001 in S2 IB). Body height was positively correlated with N2 latencies at all stimulation sites and for both stimulation protocols except S2 IB (p < 0.05 in L2 IB, L5 NB & L5 IB; p < 0.01 in L2 NB & S2 NB). In addition, pain ratings consistently correlated positively with amplitudes (p < 0.05 in L2 NB, L2 IB, L5 NB, L5 IB; p < 0.01 in S2 NB & S2 IB). All correlation matrices are illustrated in Fig. 4.

## Discussion

In the present study, we provide normative values for CHEPs from three stimulation sites on the lower extremities. These stimulation sites were chosen to allow for proximal-to-distal comparisons in length-dependent small fiber neuropathies. In addition, as each site reflects a specific spinal segment (dermatome), the normative values may also facilitate diagnoses in pathologies of the lumbar cord or in radiculopathies^25,26^.

In line with previous studies, age, and height had a significant influence on CHEP parameters^11,27–29^. The effect of age has been extensively debated by other authors^27,30,31^. Interestingly, in a study on laser evoked potentials, age had a significant influence on amplitudes but not latency^27^. The authors emphasized a central mechanism of amplitude attenuation, whereas the peripheral afferent input remains unaltered^27^. In contrast, a recent study from our group showed that latency was affected by age, yet only under the IB protocol^15^.

In the present study, age exerted a significant effect under both stimulation protocols. The discrepancy between the age-mediated effect on upper and lower extremities might be explained in terms of predominant vulnerability of fibers from the lower extremities during ageing, as most neuropathies manifest first in the lower extremities^32^. Subclinical dysfunctions may then contribute to increased latencies with ageing. Furthermore, conduction length from the lower extremities is generally longer possibly potentiating any jitter introduced by slight demyelination.

Regarding length-dependency, stimulating the dorsum of the foot (L5 dermatome) yielded significantly longer N2 latencies and smaller N2P2 amplitudes than proximal (S2 or L2) stimulation. Moreover, stimulus intensity was perceived as less painful after distal stimulation. These results are readily explained by the longer peripheral conduction length, leading to temporal dispersion of the afferent volley^2^, and the proximal-to-distal gradient in skin innervation^33^. The fact that differences in latency between distal and proximal stimulation sites persist under both stimulation protocol is of clinical relevance. This possibly facilitates the detection of distally-accentuated impairments of axonal segments in length-dependent polyneuropathies.

In studies using laser- or contact heat stimulation, N2P2 amplitudes usually correlate well within subjects with ratings of pain intensity^21^. Higher NRS scores were associated with larger N2P2 amplitudes across all stimulation sites and under both stimulation paradigms. In line with the literature, females reported higher ratings to the noxious heat stimuli^34^. Sex-related effects were also observed for N2 latencies and N2P2 amplitudes. As in previous studies^15^, we again draw upon longer conduction distances in the male subjects due to significantly greater height in order to explain these findings.

Applying the IB protocol led to shorter latencies and higher amplitudes for all stimulation sites and across all age groups^15^. The latency shift and amplitude increase were in a comparable range with data acquired for the upper extremities^15^. Stimulus characteristics should be taken into account when comparing results from different laboratories^11,27^. In slight contrast to previous studies applying CHEPs to the lower extremities^11,13^, pain ratings and some amplitudes (S2 and L2 dermatome) within the older population tend to be higher in the present study. These results can be explained in terms of improved temporal and spatial summation due to a more synchronized afferent volley using IB stimulation. A similar increase in amplitudes and subjective pain ratings was demonstrated for the stimulation of cervical dermatomes^15^. In line with results from other groups, sex differences with females displaying larger N2P2 amplitudes could also be reproduced in our data set.

For CHEPs, bearing the inherent advantage of being able to control the baseline temperature, the IB protocol is well-established and the underlying mechanisms have been extensively studied^15,21,22^. Increasing the baseline temperature of stimulation shortens stimulus duration, decreases time to threshold for receptor activation and consequently leads to a more synchronized afferent volley with an improved spatio-temporal summation at central synapses^22^. Recently, we have demonstrated that using the IB protocol for the acquisition of CHEPs from the lower extremities can improve persistence of the cortical potential in a clinically meaningful manner^12^. Our findings are in line with previous studies, Lagerburg *et al*. also reported improved acquisition when increasing the baseline temperature for stimulation in cases where there was no cortical response with NB stimulation^13^. Based on these observations, IB stimulation should be preferred over conventional stimulation whenever possible^12^.

Histological studies showed that both N2P2 amplitude and N2 latency correlate well with intra-epidermal nerve fiber density^7,35,36^. However, in the clinical routine N2 latency usually emerges as the more robust readout^15,27,37^, and has therefore been proposed as a more sensitive measure of pathology^15,27^ compared to amplitude. In line with literature^15^, amplitudes in the present study were also highly variable (i.e., high standard deviation) between subjects. Amplitudes are less reproducible over time for both upper and lower extremities^12,37^, and are susceptible to attention and arousal effects^38,39^.

Currently, the diagnostic approach to a patient with suspected small fiber neuropathy usually includes bedside examination of sensory function for both mechano- and nociception^19^. Additional confirmatory tests, like quantitative sensory testing or skin biopsies are usually recommended to substantiate the clinical diagnosis^19^. CHEPs are so far not routinely used, however, would provide an objective readout of A-delta fiber function^11^. Here, we present normative values of CHEPs for the foot dorsum, a very distal and commonly affected area in small fiber neuropathies^19^, and two more proximal stimulation sites. Toe and foot involvement occur early during disease progression in many peripheral neuropathies^32^. The relative sparing of proximal sites may facilitate the monitoring of symptom progression over time in a distal-to-proximal fashion.

CHEPs do not only bear potentially high diagnostic yield in length-dependent polyneuropathies, but also in patients with non-length dependent patterns of sensory abnormalities. For the latter, CHEPs can be employed as a sensitive measure of spinal pathology, i.e. myelopathy^5,9^. Such concomitant spinal pathology cannot be detected by skin biopsies, nor be adequately localized using quantitative sensory testing^18^. Hence, CHEPs may supplement the neurophysiological test battery as a non-invasive, objective and clinically applicable technique.

## Limitations

A major limitation of this study is the collection of normative data at only one center. Therefore, the use of the acquired normative values for CHEPs in lower extremities is limited to clinical sites using the exact same CHEP acquisition equipment. This makes the generalizability of the data weaker compared to multicenter normative data sets.

## Conclusion

In this study we provide normative values for the acquisition of CHEPs from lower extremities in a large cohort of healthy subjects across different age groups. Age, height and sex have substantial impact on the latency and amplitude of CHEPs. Latencies exhibit length-dependency allowing for an appropriate diagnosis of a proximal-distal gradient in peripheral neuropathies.

Increasing stimulation intensity markedly shortens latencies and increases amplitudes through reduced signal dispersion along the afferent fibers. This comprehensive set of normative values will improve the neurophysiological diagnosis of patients with small fiber neuropathies or neuropathic pain conditions affecting the lower extremities.
